# Legume intake on gut microbiome and glycemia in type 2 diabetes management: narrative review

**DOI:** 10.3389/fnut.2026.1785186

**Published:** 2026-04-01

**Authors:** Md. Altaf Hossain, Dominic Agyei, Andrew N. Reynolds, Biniam Kebede

**Affiliations:** 1 Department of Food Science, University of Otago, Dunedin, New Zealand; 2 Department of Medicine, University of Otago, Dunedin, New Zealand; 3 Centre of Research Excellence, Riddet Institute, Palmerston North, New Zealand; 4 Department of Applied Food Science and Nutrition, Chattogram Veterinary and Animal Sciences University, Chattogram, Bangladesh; 5 School of Chemistry, Monash University, Clayton, VIC, Australia; 6 Department of Food Science, University of Guelph, Guelph, ON, Canada

**Keywords:** diabetes management, dietary guidance, glycemic control, gut microbiome, legume consumption

## Abstract

Legumes are rich in dietary fiber, plant proteins, micronutrients, and bioactive compounds, offering a sustainable and affordable addition to the diet. However, the extent to which legume-induced modulation of the gut microbiota contributes to glycemic regulation in type 2 diabetes (T2D), relative to microbiota-independent physiological mechanisms, remains insufficiently defined. This narrative review synthesizes current evidence on legume-based interventions and their effects on gut microbiota composition and function in relation to glycemic control. Relevant studies were identified through structured searches of MEDLINE and the Cochrane Central Register of Controlled Trials via Ovid, complemented by manual screening of reference lists. Seventeen studies (three human and 14 animal trials) were considered in this review. Human studies report that consuming legumes is associated with improved glucose tolerance and blood glucose levels, although effects on microbiota composition are variable and modest. Conversely, animal studies demonstrate improvements in insulin sensitivity, glucose tolerance, and microbial diversity with higher dose legume interventions. Legume consumption has been associated with enrichment of beneficial microbial taxa, such as *Bifidobacterium*, *Akkermansia*, *Ruminococcus*, and *Bacteroides*, as well as increased concentrations of microbial metabolites such as short-chain fatty acids (SCFAs). These microbial features are implicated in metabolic pathways relevant to insulin signaling and glycemic regulation; however, current human evidence does not establish that microbiota alterations causally mediate glycemic improvements. Well-designed, adequately powered clinical studies incorporating functional microbiome analyses and formal mediation approaches are required to clarify microbiota-dependent and microbiota-independent mechanisms.

## Graphical Abstract

**Figure:**
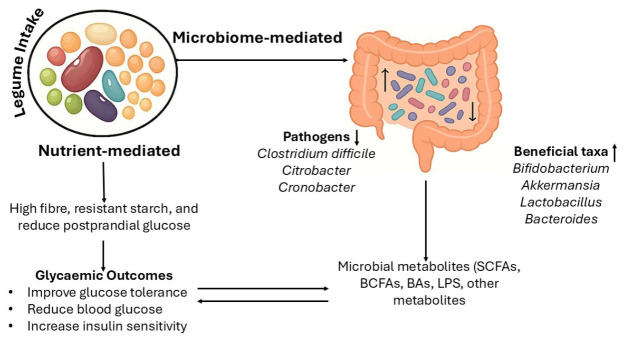
Physiological pathways mediated by legume intake.

## 1 Introduction

Type 2 diabetes is a prevalent metabolic disorder characterized by chronic hyperglycemia resulting from insulin resistance (IR) and/or impaired insulin secretion. Lifestyle modifications, particularly dietary changes, play a pivotal role in preventing and managing T2D (1). Increasingly, evidence indicates that diet quality and composition directly affect glycemic control and overall metabolic health (2, 3). Diets rich in whole grains, fruits, vegetables, and legumes show a reduced risk of developing T2D (4–6). Among these dietary components, legumes stand out for their nutrient profile, enhancing insulin sensitivity, lowering postprandial glucose levels, and improving glycemic control (7–9). Recent research reinforces the therapeutic potential of plant-based diets, especially the beneficial effects of legumes on disease progression and glycemic control, making them a key component of dietary strategies for T2D management (8, 10). Consequently, dietary guidelines, including American Diabetes Association and Diabetes New Zealand, advocate incorporating legumes into meal plans for glycemic control (11–13).

**FIGURE 1:**
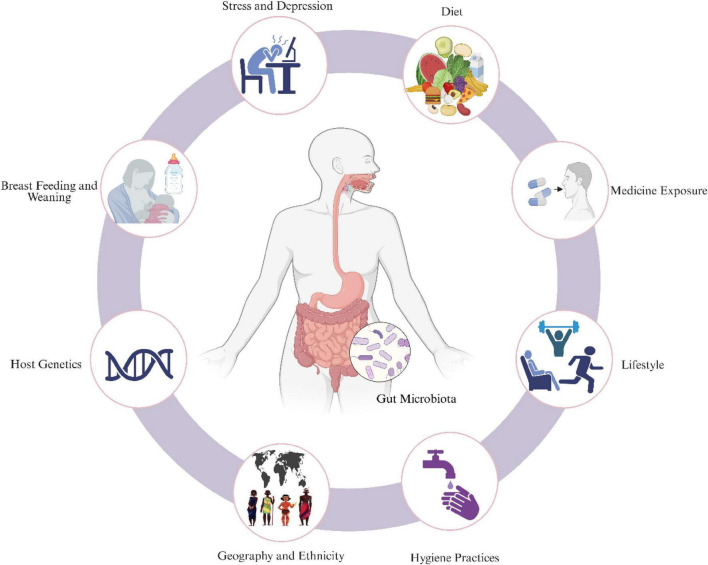
Factors affecting the host gut microbiota diversity and their functions.

Beyond the direct metabolic effects, legumes may confer additional benefits through modulation of the gut microbiota (Figure 1), the dense microbial community residing in the gastrointestinal tract that influences host metabolism, immune regulation, and inflammation (14, 15). However, the microbiome-mediated pathways linking legume consumption to improved glycemic outcomes remain insufficiently understood. Most existing reviews have addressed either broad dietary patterns (16–18) or the general gut microbiota–diabetes relationship (7, 19–21), with limited focus on legume-specific mechanisms. Notably, our recent systematic review and meta-analysis of randomized controlled trials involving ≥6 weeks of legume interventions found no studies that evaluated gut microbiome function alongside glycemic outcomes (9).

To address this gap, the present narrative review synthesizes evidence from human and animal studies examining how legume intake influences gut microbiota composition, microbial metabolites, and glycemic regulation in T2D. By integrating nutritional, microbial, and metabolic perspectives, it aims to clarify the microbiome-mediated mechanisms underlying legumes’ antidiabetic potential and to strengthen the scientific basis for incorporating legumes into evidence-based dietary strategies for T2D management.

## 2 Search strategy and review approach

This narrative review synthesizes evidence on the effects of legume and legume-based products on gut microbiota composition and glycemic outcomes in T2D. Relevant studies were identified through structured searches of MEDLINE and the Cochrane Central Register of Controlled Trials via Ovid, supplemented by manual reference screening, and included publications up to December 2025. Search terms combined keywords such as “legumes,” “pulses,” “gut microbiota,” “microbial metabolites,” and T2D-related metabolic outcomes. Peer-reviewed English-language studies were considered eligible if they investigated (i) whole legumes, isolated components, or quantifiable legume-based products, (ii) assessed gut microbiota composition and/or microbial metabolites, and (iii) reported outcomes related to glucose homeostasis. Both human and animal studies were included. Due to heterogeneity in study design, microbiome methodologies, and reported outcomes, findings were synthesized narratively.

## 3 Gut microbiota and T2D

### 3.1 Alterations of gut microbiota in T2D

People with T2D frequently have distinct gut microbiota signatures compared with healthy individuals (Table 1), marked by reduced microbial diversity and altered abundances of key bacterial taxa (22). Early studies in Denmark reported reductions in Firmicutes and Clostridia, with increases in Bacteroidetes and Proteobacteria, and significantly elevated Betaproteobacteria in people with T2D (23). Large-scale genomic study further revealed an enrichment of opportunistic pathogens (*Clostridium* species, *Escherichia coli*, *Bacteroides caccae*, and *Eggerthella lenta*) and mucin degraders (*Akkermansia muciniphila* and *Desulfovibrio* spp.), accompanied by depletion of butyrate producers such as *Roseburia*, *Eubacterium rectale*, *Clostridiales*, and *Faecalibacterium prausnitzii* (22).

**TABLE 1:** Summary of gut microbiota shifts in type 2 diabetes (T2D).

Phylum	Genus or taxa	T2D	Normoglycemic	References
Bacteria				
Firmicutes	*Lactobacillus*	↑	↓	(23, 24, 107–112)
	*Clostridium*	↓↑	↑↓	(22–24, 112, 113)
	*Roseburia*	↓	↑	(22–24, 114, 115)
	*Faecalibacterium prausnitzii*	↑↓	↑↓	(22, 24, 109, 110, 115–118)
	*Streptococcus*	↓↑	↑↓	(24, 108, 111, 117)
	*Enterococcus*	↑	↓	(108, 119)
	*Lachnospira*	↓	↑	(110, 114, 120)
	*Bulleidia*	↑	↓	(110, 120)
	*Megasphaera*	↑	↓	(120, 121)
	*Blautia*	↓↑	↑↓	(114, 122–124)
	*Anaerostipes*	↓	↑	(114, 122)
	*Dorea*	↑	↓	(117)
	*Coprococcus*	↑	↓	(124)
Bacteroidetes	*Bacteroides*	↑↓	↑↓	(22–24, 109, 111, 113, 116–118)
	*Prevotella*	↑↓	↑↓	(23, 107, 108, 110–113, 117–119)
	*Alistipes*	↑	↓	(113)
	*Parabacteroides*	↑	↓	(113)
Actinobacteria	*Bifidobacterium*	↓	↑	(107, 113)
	*Eggerthella lenta*	↑	↓	(22)
	*Collinsella*	↑	↓	(111, 117, 120)
	*Atopoium*	↓	↑	(112)
Proteobacteria	*Escherichia*/*Shigella*	↑	↓	(22, 109, 114, 121, 122)
	*Desulfovibrio*	↑	↓	(22)
	*Enterobacter*	↑	↓	(114)
	*Serratia*	↑	↓	(123)
	*Parasutterella*	↑	↓	(124)
Verrucomicrobia	Akkermansia	↑↓	↑↓	(22, 24, 108, 109, 117, 118)
Fusobacteria	*Fusobacterium*	↑↓	↑↓	(107, 121)
Arcchaea				
Euryarchaeota	Methanobrevibacter	↑	↓	(110, 114)
	Methanosphaera	↓	↑	(110)
Fungi				
Ascomycota	Alternaria	↑	↓	(110)
	Saccharomyces	↑	↓	(110)
Eukaryota				
Stramenopiles	Blastocystis	↑↓	↑↓	(110, 125)

↑, high; ↓, low in abundance; ↑↓, inconsistent.

Subsequent studies, including work in European cohorts, corroborated these findings by showing reductions in SCFAs-producing bacteria (*Roseburia*, *Eubacterium eligens*, *Bacteroides intestinalis*, and *Clostridium* spp.), and increases in *Lactobacillus* and *Clostridiales* species, and *Streptococcus mutans* increased (24). Meta-analyses and recent reviews consistently report decreased abundances of beneficial genera like *Roseburia*, *Akkermansia*, *Faecalibacterium*, *Bacteroides*, and *Bifidobacterium*, and increased levels of potentially pathogenic genera such as *Blautia*, *Fusobacterium*, and *Ruminococcus* (25–28).

Additionally, a study has identified *A. muciniphila* depletion as a hallmark of metabolic dysfunction in T2D, reflecting impaired gut barrier integrity (29). While interesting, the single study does not indicate causal associations at present with further data needed to understand the nature of the association between species-level depletion and host metabolic dysfunction. Mendelian randomization analyses further indicate potential causal associations between specific gut microbial taxa and T2D risk. Genera such as *Roseburia*, *Streptococcus*, *Ruminococcus*, *Lachnoclostridium*, *Bilophila*, and *Actinomyces* have been positively associated with increased T2D risk, whereas *Ruminococcaceae*, *Sellimonas*, *Eubacterium*, and *Oscillospira* indicate protective associations, and a potential role in reducing T2D susceptibility. Together, these findings indicate a consistent pattern in T2D characterized by a reduction in SCFAs-producing and gut barrier-supporting bacteria, accompanied by an increase of pro-inflammatory and mucin-degrading taxa.

### 3.2 Microbial metabolites implicated in T2D

**FIGURE 2:**
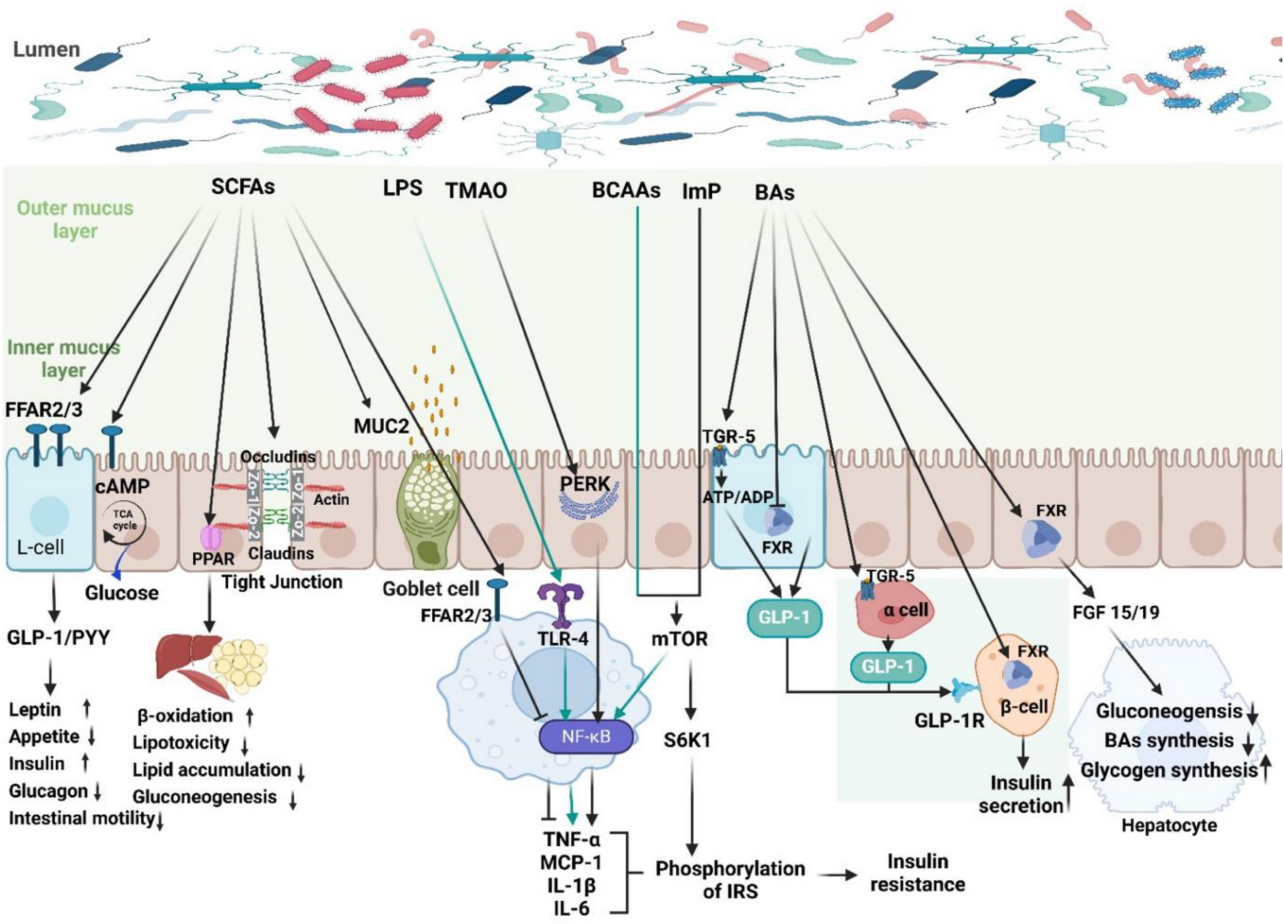
Common gut microbial metabolites and their pathways influencing glycemic regulation in type 2 diabetes (T2D). This schematic summarizes key microbiota-derived metabolites and their proposed effects on host glucose metabolism. Arrows indicate activation; blunt lines indicate inhibition. Short-chain fatty acids (SCFAs; acetate, propionate, butyrate) activate FFAR2/3 on enteroendocrine L-cells, stimulating GLP-1 and PYY secretion, enhancing insulin release, improving barrier integrity (MUC2, tight junctions), and supporting lipid oxidation via PPAR signaling. In contrast, lipopolysaccharide (LPS) activates TLR4–NF-κB signaling, increasing pro-inflammatory cytokines and promoting insulin resistance by phosphorylating IRS. Imidazole propionate (ImP) and branched-chain amino acids (BCAAs) activate mTOR/S6K1 pathways, impairing insulin signaling. Trimethylamine N-oxide (TMAO) is associated with metabolic dysfunction. Microbiota-modified bile acids signal through FXR and TGR5, regulating GLP-1 secretion, hepatic gluconeogenesis, and glycogen synthesis.

Beyond compositional changes, functional alterations in microbial metabolism contribute substantially to T2D pathophysiology (Figure 2). The gut microbiota produces a wide array of metabolites, including SCFAs, branched-chain amino acids (BCAAs), secondary bile acids (BAs), LPS, and imidazole propionate (ImP), that influence glucose homeostasis and insulin signaling (28).

#### 3.2.1 Role of SCFAs

Short-chain fatty acids, primarily acetate, propionate, and butyrate, influence glycemic regulation through multiple mechanisms, including pathways involved in insulin secretion, improving insulin sensitivity, stimulating intestinal gluconeogenesis, increasing energy expenditure, reducing fat deposition, and mitigating inflammation (30). Their metabolic effects extend beyond simple concentration-dependent actions and depend on tissue-specific metabolism, receptor distribution, and dynamic metabolic flux.

Following colonic production, SCFAs enter the portal circulation, where their physiological fate diverges. Butyrate is preferentially utilized by colonocytes as their primary energy source and regulates gene expression via histone deacetylase (HDAC) inhibition, supporting epithelial integrity and anti-inflammatory signaling (31). Propionate is largely extracted by the liver, contributing to gluconeogenic and lipid regulatory pathways (32), whereas acetate largely escapes hepatic metabolism to reach systemic circulation. There, it serves as a substrate for intracellular energy metabolism through a non-glycemic, non-insulinaemic pathway, allowing dietary fiber to provide energy without directly elevating blood glucose or stimulating insulin secretion (33).

The metabolic actions of SCFAs are mediated partly through activation of free fatty acid receptors (FFAR2 or FFAR3), whose expression varies across intestinal cells, adipocytes, immune cells, and peripheral tissues. This tissue-specific receptor distribution contributes to divergent physiological outcomes. In enteroendocrine L cells, SCFA binding influences the secretion of peptide YY (PYY) and glucagon-like peptide-1 (GLP-1), which reduce appetite, increase insulin secretion, delay gastric emptying, and suppress glucagon production (34–36). In adipose tissue, FFAR2 activation may modulate lipid storage and insulin sensitivity, while in cytokine production (37, 38). Thus, SCFAs effects are shaped not only by concentration but also by receptor localization and tissue-specific signaling context.

In addition to receptor-mediated pathways, SCFAs can regulate host metabolism via intracellular mechanisms. Propionate and butyrate promote intestinal gluconeogenesis (IGN) through cyclic 3’,5’-adenosine monophosphate (cAMP)-dependent pathways and gut–brain neural circuits, contributing to improved insulin sensitivity and glucose homeostasis (39). SCFAs also modulate gene expression through peroxisome proliferator-activated receptors (PPARs), promoting lipid oxidation, reducing lipotoxicity, minimizing lipid accumulation, and reducing gluconeogenesis in the liver, adipose tissues, and muscle (40–42).

At the intestinal level, butyrate and propionate enhance epithelial barrier integrity by upregulating tight junction proteins (occludin, claudin-1, ZO-1) and mucin-2 (MUC2), enhancing mucus production and maintaining intestinal integrity (43–45). They also exhibit anti-inflammatory properties by suppressing pro-inflammatory cytokines and chemokines, including tumor necrosis factor-alpha (TNF-α), CC chemokine ligand 5 (CCL5), monocyte chemoattractant protein-1 (MCP-1), and interleukin-6 (IL-6) (46, 47).

Despite these beneficial mechanisms, SCFA effects are dose, source, and context dependent. Exogenous propionate administration has been reported to acutely stimulate glucagon and fatty acid-binding protein 4 (FABP4), potentially increasing hepatic glucose production and impairing insulin sensitivity under certain metabolic conditions (48). Similarly, elevated systemic acetate exposure has been associated with enhanced glucose-stimulated insulin secretion and lipogenesis in some experimental models, potentially promoting hyperinsulinaemia and adiposity (49). Such paradoxical findings may reflect supraphysiological systemic exposure that bypasses normal colonic production gradients and portal metabolic processing.

Emerging evidence suggests that dynamic metabolic flux, encompassing colonic production rates, portal absorption, hepatic extraction, tissue uptake, and intracellular utilization, may be more biologically informative than absolute circulating SCFAs concentrations (50, 51). Therefore, endogenously produced SCFAs derived from fiber fermentation, generated within physiological gradients and integrated into host metabolic networks, are more likely to confer metabolic benefits than isolated or supraphysiological supplementation. Recognizing these physiological distinctions provides a more mechanistically complete framework for understanding SCFAs-mediated regulation of glycemia in type 2 diabetes.

#### 3.2.2 Role of BCAAs

Branched-chain amino acids, including leucine, isoleucine, and valine, are implicated in T2D development through mechanisms involving disrupted insulin signaling, mitochondrial dysfunction, ectopic fat deposition, and chronic inflammation (52). Elevated BCAAs activate the mammalian target of rapamycin (mTOR) complex 1, subsequently upregulate the ribosomal protein S6 kinase (S6K1), and lead to phosphorylation of insulin receptor substrate 1 (IRS-1), impairing downstream insulin signaling and reducing glucose uptake in muscle and adipose tissue, thereby contributing to IR (53).

Branched-chain ketoacids (BCKAs), metabolic intermediates of BCAAs, impair mitochondrial function, exacerbating IR and reducing insulin sensitivity (54). Elevated BCAAs and BCKAs also stimulate the release of inflammatory cytokines, such as TNF-α and IL-6, through mTOR activation and other pathways (55, 56). Chronic inflammation from these pathways disrupts insulin receptor signaling, further exacerbating IR. Additionally, BCAAs upregulate genes involved in fatty acid synthesis, that can lead to ectopic fat accumulation in non-adipose tissues, such as the liver and muscle. This ectopic fat contributes to lipotoxicity, mitochondrial dysfunction, and IR (57).

#### 3.2.3 Role of secondary BAs

The gut microbiota, including species such as *Clostridium*, *Bacteroides*, *Bifidobacterium*, *Lactobacillus*, and *Enterococcus*, transform primary BAs such as cholic acid (CA), chenodeoxycholic acid (CDCA), α-Muricholic Acid (α-MCA), and β-MCA into secondary BAs, including deoxycholic acid (DCA), lithocholic acid (LCA), ursodeoxycholic acid (UDCA), ω-MCA, and Dehydroxylated MCA (58). Secondary BAs act as natural ligands for nuclear and membrane receptors, such as the Farnesoid X receptor (FXR) and the Takeda G protein-coupled receptor 5 (TGR-5), which are expressed in various tissues, including the intestine, liver, kidney, glands, and adipose tissue. Activation of TGR-5 by secondary BAs in intestinal L-cells and pancreatic α-cells stimulates the secretion of glucagon-like peptide-1 (GLP-1), which enhances insulin secretion from pancreatic β-cells, thereby promoting glucose homeostasis and improving T2D mellitus (59–61). Secondary BAs also act on FXR in intestinal enterocytes, leading to increased fibroblast growth factor 19 (FGF19) secretion in humans (or FGF15 in rodents). FGF19/15 enters the liver via the bloodstream, where it reduces hepatic gluconeogenesis and bile acid synthesis while enhancing glycogen synthesis (60, 62, 63). Interestingly, inhibition of FXR activity in intestinal L-cells increases GLP-1 secretion, further improving glucose metabolism and reducing IR (64).

#### 3.2.4 Role of LPS

Lipopolysaccharide, or endotoxin, a component of the outer membrane of Gram-negative bacteria, is a potent inflammatory mediator that contributes to the pathophysiology of metabolic disorders, including T2D. The mechanisms involve systemic inflammation, insulin resistance, and β-cell dysfunction. LPS binds to Toll-like receptor 4 (TLR4) on the surface of immune and non-immune cells, including macrophages, adipocytes, and hepatocytes, triggering the NF-κB signaling pathway (65). This activation leads to the release of pro-inflammatory cytokines, including TNF-α, MCP-1, interleukin-1β (IL-1β), and interleukin-6 (IL-6) (66). These cytokines impair gut barrier integrity by downregulating tight junction proteins, thereby increasing intestinal permeability and promoting systemic inflammation (67). Furthermore, elevated levels of pro-inflammatory cytokines interfere with insulin signaling by inducing abnormal phosphorylation of insulin receptor substrates (IRS), compromising downstream signaling pathways necessary for glucose uptake in muscle and adipose tissues (53). This disruption exacerbates insulin resistance. Chronic inflammation also damages pancreatic β-cells, impairing insulin secretion (68, 69). Inflammatory mediators downregulate pancreatic duodenal homeobox-1 (PDX1), a gene critical for β-cell function and insulin production (70). The cumulative effects of systemic inflammation, insulin resistance, and β-cell dysfunction disrupt glucose homeostasis, exacerbating metabolic dysfunction and accelerating the progression of T2D.

#### 3.2.5 Other microbial metabolites

Imidazole propionate, a microbial metabolite derived from histidine, contributes to the pathophysiology of T2D by promoting IR and inflammation. Elevated ImP levels activate the p38γ/p62/mTORC1 signaling pathway, leading to the phosphorylation of S6K1, which induces abnormal serine phosphorylation of IRS. This modification impairs the PI3K/AKT signaling pathway, crucial for glucose uptake in skeletal muscle and adipose tissue (71). Additionally, ImP exacerbates intestinal inflammation and compromises gut barrier integrity, intensifying systemic low-grade inflammation, a hallmark of T2D progression (72).

Trimethylamine-N-oxide (TMAO), another gut microbiota-derived metabolite, exacerbates IR by activating the Protein Kinase RNA-like Endoplasmic Reticulum Kinase (PERK) pathway. PERK activation triggers NF-κB-mediated release of pro-inflammatory cytokines such as TNF-α and IL-6, which induce serine phosphorylation of IRS, impairing insulin signaling (73). Furthermore, TMAO elevates reactive oxygen species (ROS), disrupting PI3K/AKT pathway function and reducing glucose uptake in peripheral tissues (74). These combined effects underscore TMAO’s role in the metabolic dysregulation observed in T2D.

## 4 Effects of legume intake on gut microbiota and glycemia

Legume consumption has been linked to improvements in glycemic outcomes and alterations in gut microbiota composition in both preclinical and clinical studies (9). Evidence indicates multiple, potentially interacting mechanisms, including microbiota-mediated metabolic effects, reduced postprandial glycemia, and bioactive compounds. However, distinguishing causality from association remains out of reach within the context of current evidence. The following subsections summarize evidence from animal and human studies, discuss translational considerations, and highlight mechanistic pathways and clinical implications.

### 4.1 Evidence from animal models

Fourteen of the seventeen included studies were conducted in rodent models, with one study in domestic cats (75) (Table 2). In these models, legume interventions have been associated with improvements in fasting blood glucose, insulin sensitivity, and body weight, alongside alterations in gut microbiota composition and microbial metabolite profiles (76–88).

**TABLE 2:** Evidence from studies of legume intake on gut microbiota and glycemic outcomes.

Study ID, country	Design and duration	Participants	Intervention types, dose, format	Control	Gut microbiota measures (method)	T2D-related outcomes	Key findings
Feng et al. (92), China	RCT, 16 weeks	55 T2D (35–75 years)	White common bean extract (1.5 g)	1.5 g of maltodextrin	Fecal microbiota (16S rRNA)	HbA1c, FBG, PBG, OGTT, HOMA-IR	• Improved blood glucose •α-diversity did not change significantly, and β-diversity was significantly different between the groups • Family: ↓*Enterobacteriaceae* • Genus: ↑*Bifidobacterium*, *Adlercreutzia;* ↓ *Citrobacter, Cronobacter*
Lambert et al. (93), Canada	RCT, 12 weeks	50 Overweight and obese adults (age 18–70 years; BMI 25–38 kg/m^2^)	Yellow pea fiber (15 g/d)	Placebo	Fecal microbiota (qPCR)	HbA1c, plasma insulin, plasma glucose, OGTT, BW	• Improved significant glucose tolerance • Not significantly alter the gut microbiota composition
Wu et al. (94), Singapore	RCT, 16 weeks	127 pre-diabetic (age 45–75; BMI 19.5–32.0 kg/m^2^; FBG > 5.5 ↑mmol/l and < 7.0 mmol/l)	Mixed beans, red kidney beans or chick peas, 100 g per meal	Meat (chicken or fish)	Fecal microbiota (shotgun illumina sequencing)	HbA1c, FBG, insulin, HOMA-IR	• HbA1c decrease • Genus: ↑*Bifidobacterium*; ↓*Ruminococcus*, *Bacteroides*, *Bilophila* • Species: ↑*Eubacterium rectale*, *Roseburia faecis*, *Roseburia hominis*; *Ruminococcus gnavus*, *Ruminococcus torques*, *Ruminococcus lactaris*,*Bacteroides massiliensis*, *Bacteroides stercoris*, *Bacteroides ovatus*, *Bilophila wadsworthia*
Bai et al. (76), China	RCT, 4 weeks	56 male Wistar rats (200 ± 20 g)	100–400 mg/kg bw of extracted polysaccharides from small black soybean	Distilled water	Ceacum microbiota (16S rRNA)	FBG, HOMA-IR	• Improved FBG and reduced insulin resistance •α-diversity increased, and β-diversity was different among the groups •↑*Oscillospira*, *Ruminococcus*, *Dorea*; ↓*Mucispirillum*
Bai et al. (77), China	RCT, 4 weeks	56 male Wistar rats (200 ± 20 g)	100–400 mg/kg bw of extracted polysaccharides from red kidney bean	Distilled water	Ceacum microbiota (16S rRNA)	FBG, serum insulin, HOMA-IR	• Significantly reduced FBG, HOMA-IR levels and improve insulin resistance •α-diversity increased • Genus: ↓*Oscillospira*, *Mucispirillum*; ↑*Treponema*, *Bacteroides*, *Phascolarctobacterium*, *Succinivibrio*, *Blautia*
Chen et al. (78), China	RCT, 4 weeks	18 STZ induced diabetic Wistar male rates (180–220 g)	White hyacinth bean polysaccharides (100 mg/kg bw)	Distilled water	Fecal microbiota (16S rRNA), cecum SCFAs	FBG, BW	• Significantly reduced FBG •α-diversity increased and β-diversity was different among the groups • Phylum: Firmicutes to Bacteroides ratio increased • Genus: ↑*Allobaculum*, *Eubacterium*, *Anaerobiospirillum* and *Holdemania*
Eslinger et al. (79), Canada	RCT, 6 weeks	100 male Sprague-Dawley rats HFD induced obesity (5 weeks)	Yellow pea fractions (30% by bw)	Normal diet	Cecal microbiota (16S rRNA)	FBG, FBI, OGTT, BW	• Improve blood glucose and insulin sensitivity • Lower body weight and body fat%) • Phylum: ↓Firmicutes • Species: ↓ *Clostridium leptum* (cluster IV)
Hashemi et al. (80), Canada	RCT, 4 weeks	32 Male Sprague Dawley rats (8 weeks)	Raw and cooked Pea fiber	High and low-fat diet	Fecal microbiota (16S rRNA), and SCFAs	OGTT, plasma insulin	• Improve glycemic outcomes • Improve microbial diversity • SCFAs: ↑acetate and propionate • Phylum: ↓Bacteroidetes; ∼ Firmicutes • Family: ↑Lachnospiraceae and Prevotellaceae; ↓ Porphyromonadaceae
Hou et al. (81), China	RCT, 12 weeks	Male C57BL/6J mice	Mung bean seed coat	NCD and HFD	Fecal microbiota (16S rRNA)	OGTT, BW, GBG, FSI, HOMA-IR	• Improved FBG and insulin resistance •α-diversity did not change significantly, but β-diversity was distinct among the groups • Phylum: ↑Bacteroidetes, Verrucomicrobia, and Actinobacteria; ↓Firmicutes, Proteobacteria, and Deferribacteres • Family: ↓ *unclassified_f_Ruminococcaceae*; ↑ *Lachnospiraceae_ NK4A136_group*, and *norank_f_Muribaculaceae* • Genus: ↓*Blautia*, *Ruminiclostridium_9*, and *Bilophila*; ↑ *Akkermansia*
Li et al. (80), China	RCT, 4 weeks	5-week-old Sprague-Dawley rats (120–150 g)	Chickpea extract (3 g/kg bw)	NCD and HFD	Fecal microbiota (16S rRNA), SCFAs	FBG, insulin, HOMA-IR, BW	• Improved insulin resistance • Increased microbial richness • SCFAs: ↑acetic, propionic, and butyric acid • Phylum: ↑Bacteroidetes/Firmicutes • Family: ↑Alcaligenaceae, Bacteroidaceae, Enterococcaceae, and Turicibacteraceae • Genus: ↑ *Bacteroides*, *Blautia*, *Alcaligenaceae*, *Sutterella*, *Enterococcus*, *Turicibacter*, and *Dorea*
Liu et al. (83), China	RCT, 8 weeks	54 C57BL/6 male mice (4 weeks)	Pea albumin (1.5 g/kg bw)	NCD and HFD	Fecal microbiota (16S rRNA)	FBG, FBI, OGTT, BW, BF%	• Improved glucose tolerance and insulin sensitivity • Reduced body weight •α-diversity and β- diversity increased • Phylum: ↑Bacteroidetes, Desulfobacterota and Verrucomicrobia; ↓Firmicutes • Genus: ↑*Blautia, Bacteroidea*, *norank-f-lachnospiraeceae*, *Akkermansia* and *Parabacteroides*; ↓*Lactobacillus*, *Staphylococcus*, *Lactococcus*
Mo et al. (75), China	RCT, 4 weeks	40 spayed or neutered domestic adult cats (3.07 ± 0.36 years)	Pea starch 30% of total diet	Corn starch 30% of total diet	Fecal microbiota (16S rRNA), SCFAs	BG, Insulin, BW	• Lower the glucose and insulin level • Increased microbial diversity • No significant changes in SCFAs • Genus: ↑*Ruminococcus*, *norank_f__Eubacterium_coprostanoligenes_group* and *Blautia*
Monk et al. (84), Canada	RCT, 12 weeks	HF induced male C57BL/6 mice (4 weeks)	Cooked navy bean powder + HF diet 15.7% by feed weight	HF (isocaloric with intervention)	Fecal microbiota (16S rRNA), SCFAs	HOMA-IR	• Improved insulin sensitivity •↑α-diversity and β- diversity changed • Phylum: ↑Proteobacteria • Genus: ↑*Prevotella*, *Sutterella*, *Allobaculum* and *Coprococcus*;↓*Lactococcus, rc4–4, Parabacteroides* and *Adlercreutzia* • Species: ↑*Akkermansia muciniphila;*↓*Ruminococcus gnavus* • SCFAs: ↑acetic acid, propionic acid, butyric acid, valeric acid
Sánchez-Tapia et al. (85), Mexico	RCT, 8 weeks	32 male Wistar rats (6 weeks)	HFD + black bean	HFD	Fecal microbiota (16S rRNA), SCFAs	FBG, FBS, OGTT, BW	• Reduced body weight and body fat% • Low level of glucose and insulin •↑α-diversity and β- diversity changed • Phylum: ↑Cyanobacteria; ↓Proteobacteria • Genus: ↑*Ruminococcus*, *Coprococcus* and *Prevotella* • Species: ↑ *Ruminococcus bromii*, *Clostridium eutactus*, *Ruminococcus Callidus*, *Ruminococcus flavefaciens* • SCFAs: ↑butyrate; ↓acetate, propionate
Sun et al. (86), China	RCT, 4 weeks	STZ induced diabetic male Sprague-Dawley rats (200 ± 20 g)	HFD + Black bean husk (400 mg/kg bw)	HFD	Cecal microbiota (16S rRNA)	FBG, insulin, OGTT, HOMA-IR, BW	• Improved glycemic outcomes and insulin sensitivity • Phylum: ↑ Firmicutes and Verrucomicrobias; ↓ Actinobacteria • Genus: ↑*Akkermansia*, *Coprococcus*, *Bacteroides*, and *Phascolarctobacterium*; ↓ *Bifidobacterium*
Tan et al. (87), USA	RCT, 6 weeks	16 male C57BL/6J mice	HFD + 20% black turtle bean	HFD	Fecal microbiota (16S rRNA)	HOMA-IR, FBI, OGTT	• Improved insulin sensitivity significantly • No significant different in insulin level • No change in α-diversity • Phylum: ↑Bacteroides; ↓Firmicutes, Actinobacteria, Verrucomicrobia • Genus: ↑ *Lachnospiraceae NK4A136* group, *Ruminococcus 1*; ↓ *Blautia*, *Clostridium sensu stricto1*, *Erysipelatoclostridium*, *Romboutsia*, *Turicibacter*
Yu et al. (88), China	RCT, 3 weeks	60 male C57BL/6J mice (3 weeks)	Soybean peptide and white kidney bean extract	Standard diet	Fecal microbiota (16S rRNA) SCFAs	FBG, BW, HbA1c, insulin, OGTT	• Significantly reduced HbA1c level and increased insulin level • Decreased FBG level •↑α-diversity and β- diversity changed • Phylum: ↑Bacteroidetes; ↓Firmicutes • Genus: ↑*Bifidobacterium*, *Ruminococcus*, *Akkermansia*; ↓*Dorea*, *Anaerostpes*

RCT, randomized controlled trial; T2D, type 2 diabetes; FBG, fasting blood glucose; FBI, fasting blood insulin; HOMA-IR, Homeostatic Model Assessment of Insulin Resistance; bw, body weight; HFD, high fat diet; OGTT, oral glucose tolerance test; HbA1c, glycated hemoglobin; SCFAs, short chain fatty acids; NCD, normal low-fat control diet; STZ, streptozotocin; BD, basal diet.

Fermentable fibers and resistant starches in legumes were associated with increased relative abundance of SCFA-producing taxa such as *Bacteroides*, *Bifidobacterium*, *Akkermansia*, *Prevotella*, and *Ruminococcus*, often accompanied by greater microbial diversity (77, 82–84, 86, 88). However, increased microbial diversity should not be interpreted as uniformly beneficial, as functional output, such as metabolite production and pathway activity, is likely more relevant to metabolic outcomes than diversity metrics alone (89). Elevations in gut microbiota-derived metabolites, including SCFAs such as acetate, propionate, butyrate, and valerate, were reported in parallel with markers of improved intestinal barrier integrity, GLP-1 secretion, and glucose homeostasis (80, 82, 84, 85). Importantly, these findings reflect controlled experimental conditions in animal models and do not provide evidence of microbial mediated metabolic improvements. Rather, they provide mechanistic plausibility for microbiota-related pathways.

Short-chain fatty acid responses in the available animal studies were context dependent. For example, black bean supplementation increased butyrate but reduced acetate and propionate, reflecting microbial cross-feeding and substrate preference (85) or chance or selective findings. Similarly, the metabolic effects of SCFAs are influenced by dose, site of production (cecum vs. colon), and host metabolic state (90). Several animal studies also reported shifts in the Firmicutes/Bacteroidetes (F/B) ratio, generally indicating a reduction following legume intake, though the relevance of this metric as a biomarker of metabolic health remains debated (81, 83). Furthermore, the transient or sustained nature of such a shift in ratio is unknown, given the relatively short duration of studies identified. Contemporary microbiome research indicates that this ratio varies substantially across populations, sequencing platforms, analytical pipelines, and disease contexts, limiting its reliability as a standalone indicator of metabolic status (91). Therefore, observed changes in the F/B ratio should be interpreted as descriptive compositional shifts of uncertain duration and effect, rather than definitive markers of metabolic improvement. Overall, animal studies indicate biologically plausible links between legume intake, microbial alterations, and metabolic adaptations. However, high dietary doses and species-specific physiology prevent direct extrapolation to human dietary contexts.

### 4.2 Evidence from human studies

Only three human intervention studies with 232 participants were identified that examined legume intakes in relation to gut microbiome and glycemic outcomes (92–94). Interventions included white common bean extract (92), yellow pea fiber (93), and mixed beans or chickpeas (94), over 12–16 weeks. All trials report modest improvements in fasting glucose, insulin sensitivity, or HbA1c. Feng et al. (92) observed increases in *Bifidobacterium* and *Adlercreutzia* alongside reductions in *Enterobacteriaceae* (92). Wu et al. (94) reported increases in SCFAs-producing taxa (*Bifidobacterium*, *E. rectale*, *Roseburia* spp.) and reductions in pro-inflammatory taxa (*Bilophila*), along with decreased HbA1c (94). In contrast, Lambert et al. (93) found improved glucose tolerance without significant microbiota changes (93).

However, these findings warrant deeper critical interpretation. First, it remains unclear whether reported glycemic improvements were independent of changes in body weight, energy intake, or broader dietary composition. In one study, participants experienced small weight loss or altered energy intake (93), which could partially or fully explain observed metabolic effects, suggesting that benefits may not be entirely microbiota-mediated. Second, the temporal relationship between microbial shifts and metabolic improvements was not established. Microbiota and metabolic markers were typically measured at baseline and study endpoint, preventing assessment of whether microbial alterations preceded, coincided with, or followed glycemic changes.

Additionally, small sample sizes, differences in baseline microbiota and dietary background, and heterogeneous microbiome assessment methods constrain mechanistic insight. Two trials used 16S rRNA sequencing, which provides limited functional resolution and does not quantify microbial metabolites directly. Observed microbial shifts were generally modest and inconsistently associated with glycemic outcomes, highlighting that the mechanisms underlying the metabolic improvements remain unresolved.

**FIGURE 3:**
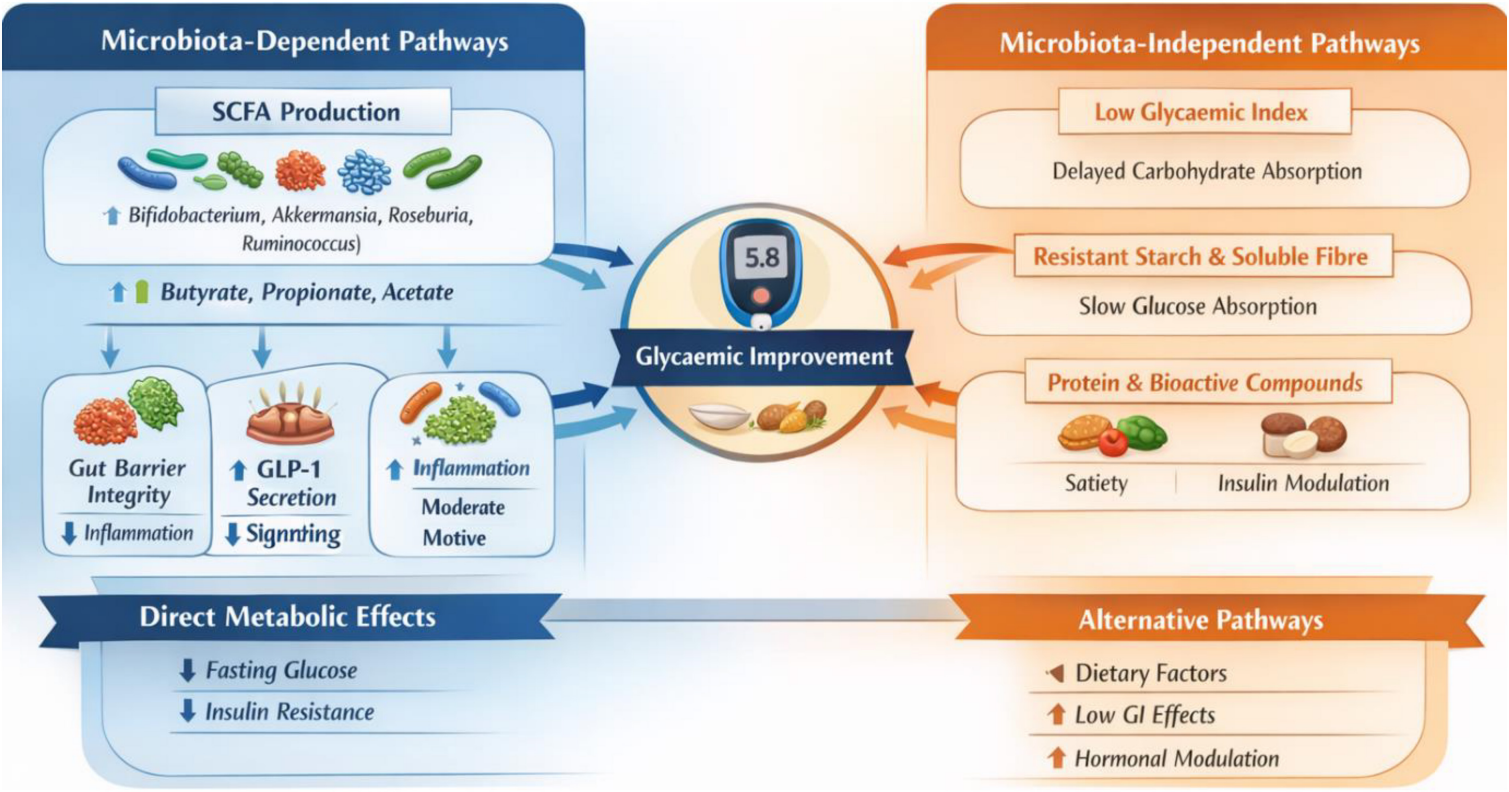
Conceptual framework: microbiota-dependent vs. microbiota-independent mechanisms of legume effects on glycemia.

Therefore, while human evidence supports associative links between legume intake, microbiota modulation, and improved glycemic markers, it does not yet establish causality or clarify whether microbial changes are primary mediators. Glycemic benefits likely arise from multiple interacting mechanisms, including delayed carbohydrate absorption, increased dietary fiber and protein, hormonal modulation, and potentially microbiota-derived metabolites (Figure 3).

### 4.3 Translational considerations

Several factors limit direct translation of preclinical findings to humans. Animal studies typically use dietary doses representing 15%–40% of total intake (75, 79, 84, 87), which exceeds usual human consumption. Baseline microbiota composition, bile acid pool, and metabolic dynamics differ substantially between rodents and humans, influencing microbial responses and downstream metabolite production. Variability in sequencing approaches, taxonomic resolution, and functional assays across human studies further complicates cross-study comparisons and translational interpretation.

Although legume-induced shifts in gut microbiota composition are frequently associated with improvements in glycemic outcomes, the current body of evidence does not establish clear cause and effect. In some studies, glycemic improvements have occurred without significant microbial changes (79, 93), whereas in others, microbiota alterations were not consistently accompanied by metabolic benefits (75, 85, 94). These observations suggest that microbiota changes may function as mediators, modulators, parallel adaptations, or secondary markers of dietary modification. Distinguishing among these possibilities requires integrated mediation modeling, metabolomics, and functional microbiome profiling, which remain limited in existing trials. Accordingly, preclinical findings should be interpreted as demonstrating mechanistic plausibility rather than established clinical causality.

### 4.4 Implications for T2D management

Increasing legume intakes represent a nutritionally robust, affordable, and culturally acceptable strategy for T2D management (12), with evidence supporting improvements in fasting glucose, HbA1c, and insulin sensitivity (76, 77, 86, 88, 92, 94–96). These benefits likely arise from multiple complementary mechanisms, including microbiota-dependent pathways, such as increased production of SCFAs, enrichment of beneficial taxa including *Bifidobacterium*, *Akkermansia*, and *Roseburia*, and modulation of gut barrier integrity and enteroendocrine signaling. Concurrently, microbiota-independent mechanisms include reduced postprandial glycemia, resistant starch content, higher protein levels, delayed carbohydrate absorption, and direct metabolic and hormonal effects (Figure 3).

Although microbiota-mediated mechanisms are biologically plausible and somewhat supported by emerging human data, current evidence does not conclusively establish causal mediation in clinical populations. Therefore, legumes should be considered within a multifactorial dietary framework for T2D management, integrating direct metabolic, hormonal, and potentially microbiota-related effects, while also aligning with plant-forward dietary recommendations (1) and broader sustainability goals (97).

## 5 Challenges and considerations

The metabolic response to legume intake in T2D management appears to vary across individuals and may be influenced by host genetics, baseline gut microbiota composition, and dietary background such as average dietary fiber intake. Some studies suggest that individuals with higher baseline abundance of beneficial taxa may exhibit greater metabolic responsiveness (98); however, predictive microbial signatures have not been validated in large human cohorts (99) of long-term randomized controlled trials. Genetic factors, such as differences in enzyme secretion and metabolic processes, can also influence digestive capacity and metabolic response to legumes (100, 101). These microbial and genetic differences suggest that the benefits of legume intake for T2D management may not uniform across populations.

Gastrointestinal discomfort remains a common challenge associated with increasing legume consumption, particularly for those moving from low fiber intakes to much higher intakes given the fiber-density of legumes. Fermentable fibers undergo colonic fermentation, which can produce gas and transient symptoms such as bloating, flatulence, and abdominal discomfort (102, 103) with rapid increases in fiber intake. However, tolerance varies by legume type and individual sensitivity. For instance, some evidence indicates that lentils and chickpeas may be better tolerated in small amounts compared to kidney beans, which contain higher levels of fermentable fiber (104). Alongside the dietary advice to slowly increase intakes of legumes due to their high fiber density, emerging strategies to improve tolerability include the development of modified legume varieties and the application of processing techniques such as soaking, sprouting, and fermenting, which can reduce fermentable fiber content (105, 106). These considerations underscore that legume effects are context-dependent and may benefit from personalized dietary approaches.

## 6 Conclusion and future perspectives

Available evidence indicates that legume intake is associated with improvements in glycemic markers in T2D, potentially involving both microbiota-related and microbiota-independent pathways. Preclinical studies provide mechanistic insight, while human trials demonstrate modest metabolic benefits. However, existing human studies do not establish that legume-induced microbiota alterations causally mediate improvements in glycemic control. Most data are associative, and formal mediation analyses integrating metagenomics, metabolomics, and glycemic phenotyping are lacking. Future well-powered randomized controlled trials incorporating functional microbiome profiling and mediation modeling are needed to determine whether microbial metabolites act as causal mediators, contributory modulators, or secondary markers of metabolic adaptation. Additionally, examining the effects of different legume processing methods (soaking, sprouting, fermenting) will provide insights into optimizing tolerability, nutrient bioavailability, and metabolic outcomes.

Addressing these gaps will strengthen the evidence base for incorporating legumes into the daily diet as a strategy for T2D management, aligning with global health initiatives promoting sustainable, plant-based diets to mitigate the growing burden of diabetes.
